# The Relation between the Use of Antitoxin and the Occurrence of Diphtheritic Paralysis

**Published:** 1899-09-02

**Authors:** 


### Hospital Clinics and Medical Progress.

### THE RELATION BETWEEN THE USE OF ANTITOXIN AND THE OCCURRENCE OF DIPHTHERITIC PARALYSIS.

In a careful paper in last week's *Lancet* Dr. Woollacott, of the Eastern Fever Hospital, examines the subject of the relation between the use of antitoxin and the occurrence of diphtheritic paralysis. What seems to have happened is that while under the use of antitoxic serum in the treatment of diphtheria, in the London fever hospitals, the death-rate from that disease has fallen, the paralysis rate has risen. This increase of paralysis has no doubt been chiefly due to the fact that many more patients now recover from the primary disease, and thus live long enough for paralysis to show itself—patients who would formerly have died before it had a chance of appearing. But during the last two years the occurrence of paralysis has begun to diminish in frequency, and the question arises whether this is not the result of the more efficient use of antitoxin during that period. Towards the end of 1896 antitoxin began to be given in larger doses, and this rise in the dose has been maintained, and indeed increased, during the succeeding years. While before that time small doses, *e.g.*, 1,500 to 2,000 units, were common, in the early part of 1897 the minimum dose at the Eastern Hospital was 3,000 units, and later rose to 4,000 units, at which it still remains, however mild the case might be, no attempt being made to reduce the dose. Severe cases, of course, received still larger doses, and Dr. Woollacott believes that it is to the systematic employment of these comparatively large doses that the recent fall in the paralysis rate is to be attributed. In 1898 the minimum dose was always 4,000 units, and practically all the cases were injected, and in this year there was still less paralysis. The conclusions, then, at which he arrives are that, although the percentage of paralysis has increased as a whole, there is some evidence that large doses of antitoxin are more effective than small ones, both in preventing paralysis and in diminishing the mortality due to it; but that the full value of antitoxin is only obtained by using it early and in efficient doses. If this is done not only is life saved, but tedious complications are prevented, or at least are deprived of their dangerous characters.

